# Isolation Improvement of Parasitic Element-Loaded Dual-Band MIMO Antenna for Mm-Wave Applications

**DOI:** 10.3390/mi13111918

**Published:** 2022-11-06

**Authors:** Musa Hussain, Wahaj Abbas Awan, Esraa Musa Ali, Mohammed S. Alzaidi, Mohammad Alsharef, Dalia H. Elkamchouchi, Abdullah Alzahrani, Mohamed Fathy Abo Sree

**Affiliations:** 1 Department of Electrical Engineering, Bahria University Islamabad Campus, Islamabad 44000, Pakistan; 2 Department of Information and Communication Engineering, Chungbuk National University, Cheongju 28644, Korea; 3 Faculty of Aviation Sciences, Amman Arab University, Amman 11953, Jordan; 4 Department of Electrical Engineering, College of Engineering Taif University, P.O. Box 11099, Taif 21944, Saudi Arabia; 5 Department of Information Technology, College of Computer and Information Sciences, Princess Nourah Bint Abdulrahman University, P.O. Box 84428, Riyadh 11671, Saudi Arabia; 6 Department of Electronics and Communications Engineering, Arab Academy for Science, Technology and Maritime Transport, Cairo 11865, Egypt

**Keywords:** MIMO antenna, isolation improvement, compact electronics, 5G, 28 GHz

## Abstract

A dual-band, compact, high-gain, simple geometry, wideband antenna for 5G millimeter-wave applications at 28 and 38 GHz is proposed in this paper. Initially, an antenna operating over dual bands of 28 and 38 GHz was designed. Later, a four-port Multiple Input Multiple Output (MIMO) antenna was developed for the same dual-band applications for high data rates, low latency, and improved capacity for 5G communication devices. To bring down mutual coupling between antenna elements, a parasitic element of simple geometry was loaded between the MIMO elements. After the insertion of the parasitic element, the isolation of the antenna improved by 25 dB. The suggested creation was designed using a Rogers/Duroid RT-5870 laminate with a thickness of 0.79 mm. The single element proposed has an overall small size of 13 mm × 15 mm, while the MIMO configuration of the proposed work has a miniaturized size of 28 mm × 28 mm. The parasitic element-loaded MIMO antenna offers a high gain of 9.5 and 11.5 dB at resonance frequencies of 28 GHz and 38 GHz, respectively. Various MIMO parameters were also examined, and the results generated by the EM tool CST Studio Suite^®^ and hardware prototype are presented. The parasitic element-loaded MIMO antenna offers an Envelop Correlation Coefficient (ECC) < 0.001 and Channel Capacity Loss (CCL) < 0.01 bps/Hz, which are quite good values. Moreover, a comparison with existing work in the literature is given to show the superiority of the MIMO antenna. The suggested MIMO antenna provides good results and is regarded as a solid candidate for future 5G applications according to the comparison with the state of the art, results, and discussion.

## 1. Introduction

The main requirements for future 5G communication systems are high data rates, very low latency, high network capacity, wider availability, and multi-user support. To overcome these challenges, the antenna used in 5G devices plays a critical role [1]. Due to their high gain and wideband characteristics, Multiple Input Multiple Output (MIMO) antennae are thought to be the best candidates for meeting the aforementioned requirements [2]. A MIMO millimeter-wave antenna configuration offers high gain, a wide bandwidth, and high efficiency, resulting in a high data rate >20 Mbps and a low latency rate [3].

In recent years, MIMO technology has received much attention due to its numerous advantages, including increased data capacity, beam steering, and diversity. To obtain significantly better outcomes, the MIMO antenna’s various parameters have been adjusted in terms of isolation, gain, or Envelope Correlation Coefficient (ECC) and Channel Capacity Loss (CCL), etc. A variety of strategies are used to optimize these MIMO antenna characteristics [4]. The MIMO antenna’s isolation can be refined by adding an Electronic Bandgap (EBG), Retrodirective Antenna (RDA), metamaterial, Defected Ground Structure (DGS), shorting pins, or parasitic elements [5].

In the literature, a number of antenna designs are reported for millimeter bands [6,7,8,9,10,11,12], MIMO antennae for 5G communication [13,14,15,16,17,18,19,20,21], and MIMO antennae with improved isolation [22,23,24,25,26,27,28]. A single-element compact antenna for millimeter-wave dual-band applications was proposed to offer reconfigurability between 5G globally allocated bands 25 GHz and 50 GHz [6]. The approach of the phased array was also adopted to obtain a high-gain millimeter-wave antenna [7] or a simple and compact feed network array [8]. To avoid the complexity and the large size of the array, a compact antenna for 28/38 GHz has been reported for high-gain dual-band applications [9]. The Co-Planar waveguide (CPW) feed slot antenna for 28/38 GHz was reported with the advantages of easy fabrication, effective broadband performance, and low dispersion [10]. A broadband antenna operating over 28 GHz or 38 GHz or both was also reported, with a compact size and simple geometry [11,12]. The reported products have either offered a compact size or broadband with high gain. There is still a lack of high gain, as in most of the research, high gain is obtained by using an array approach or having a large size and more structural complexity.

As discussed before, a MIMO antenna can offer higher gain and a wider band to meet all the requirements discussed above for a 5G communication system [13,14]. The high data rate and large user capacity are granted by the high gain and wideband antenna [15]. In [16], a four-port MIMO system with general measurements of 100 mm × 75 mm × 0.258 mm is proposed for 28/38 GHz applications. The antenna has simple geometry but a large size as well as a narrow bandwidth of 0.7 GHz at 28 GHz. Another antenna for 28 GHz is reported in [17]. Although the reported product is minute in size (that is, 10 × 12 mm^2^), it has the demerit of low gain.

The air-filled, slot-structured antenna reported in [18] has a high gain of 6.9 dBi, operating at 28 GHz. Despite the fact that the reported product has a steep gain, it has a complex structure and high isolation. DGS is loaded to the ground plane to obtain a high gain and wideband antenna, but the literature notes that many incidents of energy are reflected back, which is not suitable and appropriate for smart device applications because microwave and millimeter-wave radiation can harm humans [19,20,21].

Mutual coupling is a key parameter of MIMO systems and plays a crucial role in obtaining a high data rate. Mutual coupling can be reduced by adopting various techniques, including DRA [22], EBG structures [23], metal strips [24], parasitic elements [25], metamaterials [26], or parasitic elements along with DGS [27]. A metamaterial-inspired DRA antenna with high isolation was reported in [22]. Although the isolation improved from −16 dB to −23 dB, there was not much improvement as compared to other reported work in the literature. In [23], double-sided EBG structures were inserted between MIMO elements to cut back the isolation from −52 dB to −60 dB. The reported antenna had a miniaturized size of 15.3 mm × 8.5 mm × 0.79 mm and high isolation, but its setback was the narrow bandwidth of 1 GHz at 28 GHz and 0.9 GHz at 38 GHz.

In [24], a metal strip was loaded between the elements of a MIMO antenna to reduce mutual coupling. The reported antenna offered a peak gain of 8.2 dBi but a narrow band ranging from 27.5 to 28.5 GHz and a complex structure. Mutual coupling by a C-shaped parasitic element was reported in [25]. The antenna offered a peak gain of 9.8 dBi but operated at 17 GHz, although it did show isolation improvement by using parasitic elements. Another product in [26] is reported for a 28/38 GHz application. The reported antenna has a small size of 25 mm × 15 mm × 1 mm, but complex geometry. A MIMO antenna for V2X communication was reported in [27] with a high isolation value of −60 dB. The antenna consisted of DGS and a parasitic element and was operational on the lower band of 5.6 GHz but is discussed here due to the technology used for mutual coupling reduction.

In this article, a compact, geometrically simple, high-gain, wideband antenna operating at 28/38 GHz is recommended for 5G applications. The four-element MIMO configuration is adopted to meet the requirements of future millimeter-wave 5G devices. In the final stage, a parasitic element is loaded between the MIMO antenna elements to cut back the mutual coupling. The remainder of the article is split as follows. In Section 2, the single element of the antenna along with the design stages, parametric analysis, and results are given. The MIMO antenna as well as the parasitic element-loaded MIMO antenna are discussed in Section 3, which also contains various results of the MIMO antenna and hardware prototype. The conclusion regarding the suggested antenna, along with references used in the literature, is provided in Section 4. A comparison table is given to compare the proposed antenna with those already published in the literature.

## 2. Single Element Wideband Antenna Operating at 28/38 GHz

### 2.1. Antenna Design

**Figure 1:**
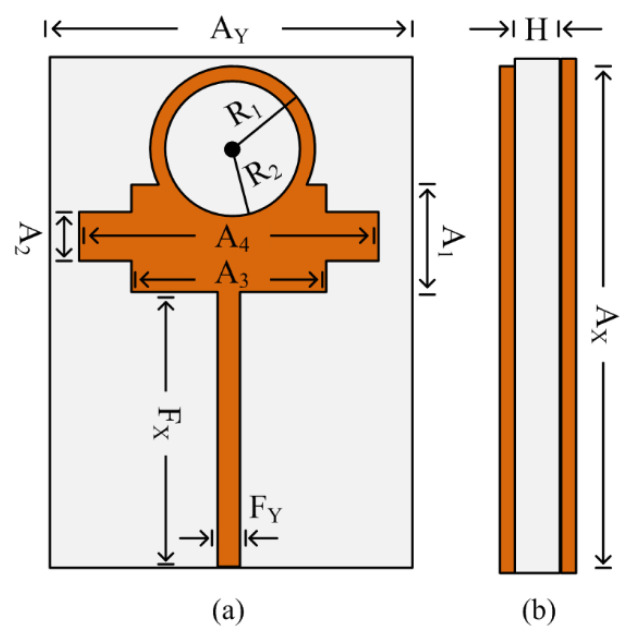
Geometrical structure of the proposed wideband millimeter-wave antenna: (**a**) top view; (**b**) side view.

Figure 1 represents the geometrical configuration of the wideband compact antenna for millimeter-wave applications. The proposed antenna is embedded over commercially available substrate material Rogers/Duroid RT5870, with a relative permittivity and a loss tangent of 2.33 and 0.0012, respectively. The geometry of the proposed antenna contains a microstrip feedline and a rectangular patch loaded with a circular stub and slots. The proposed antenna has a compact size of *A_Y_* × *A_X_* × *H* = 13 mm × 15 mm × 0.79 mm. The optimized parameters of the proposed dual-band antenna are given as follows: *A_X_* = 15; *A_Y_* = 13; *A*_1_ = 3; *A*_2_ = 1.5; A_3_ = 6; *A*_4_ = 8; *F_X_* = 8; *F_Y_* = 0.5; *R*_1_ = 2; *R*_2_ = 2.3; *H* = 0.79. All units are in millimeters (mm).

### 2.2. Antenna Design Stages

The wideband antenna suggested in this article has been designed for application at 28/38 GHz after following various design stages. Initially, the microstrip line rectangular patch antenna was engineered for use at 28 GHz. The equations below were used to determine the rectangular patch’s length (L) and width (W) [28].

(1) $$W=\frac{c}{2F\sqrt{\frac{\varepsilon r+1}{2}}}$$

L = L_eff_ + 2 Δ*L* (2)

where

(3) $$L_{\mathrm{eff}}\;=\frac{c}{2F\sqrt{\varepsilon_{\mathrm{reff}}}};\;\varepsilon_{\mathrm{reff}}=\frac{\varepsilon r+1}{2}+\frac{\varepsilon r-1}{2}\left[\frac{1}{1+12\;\frac{H}{W}}\right];\;\Delta L=0.412H\frac{\left(\varepsilon_{\mathrm{reff}}+0.3\right)\;\left(\frac{W}{H}+0.264\right)}{\left(\varepsilon_{\mathrm{reff}}-0.258\right)\left(\frac{W}{H}+0.8\right)}$$

**Figure 2:**
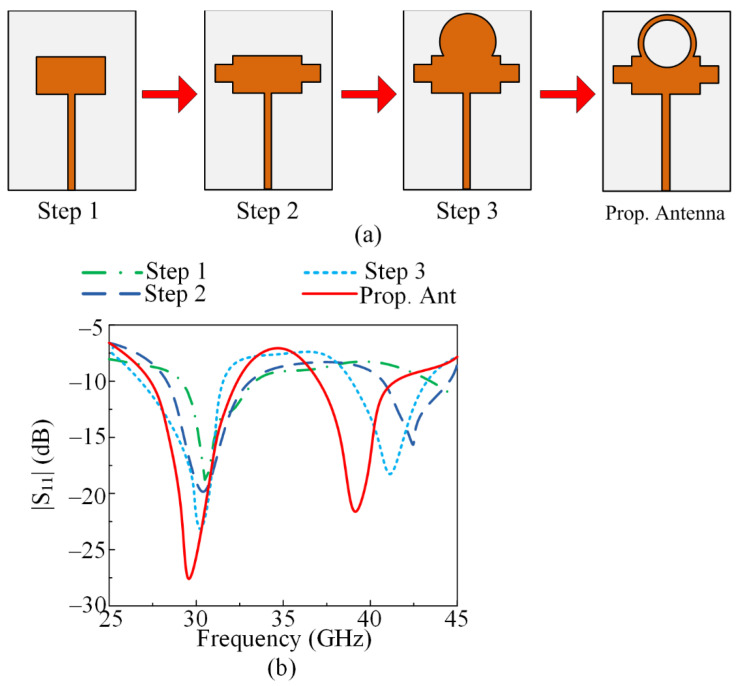
(**a**) Design evolution of proposed dual-band antenna and (**b**) S-parameter graph of various design steps.

In the next designing stage, an additional rectangular patch was inserted into the antenna, which caused the generation of second resonance along 43 GHz. The antenna after this stage resonated at 30 GHz with a return loss of −20 dB and at 43 GHz with a return loss of −15 dB. In the third stage, an additional circular shaped patch was loaded on top of the existing rectangular radiator, which improved the return loss as well as lowering the higher resonance mode at 38 GHz. In the final stage, a circular slot of radius R2 = 2.3 mm was etched from the radiator of the antenna, as given in Figure 2a, which resulted in shifts in a frequency band and improved the return loss. The resultant antenna after the final step operated at 26.5–31.5 GHz and 35–41 GHz, as depicted in Figure 2a,b. 

### 2.3. Single Element Antenna Results and Discussions

To validate the dual-band antenna simulation-based results for mm-wave applications, a hardware prototype was fabricated and measured. Various results in terms of the scattering parameters, gain, and radiation patterns are discussed below. 

#### 2.3.1. Measured and Simulated S-Parameter

**Figure 3:**
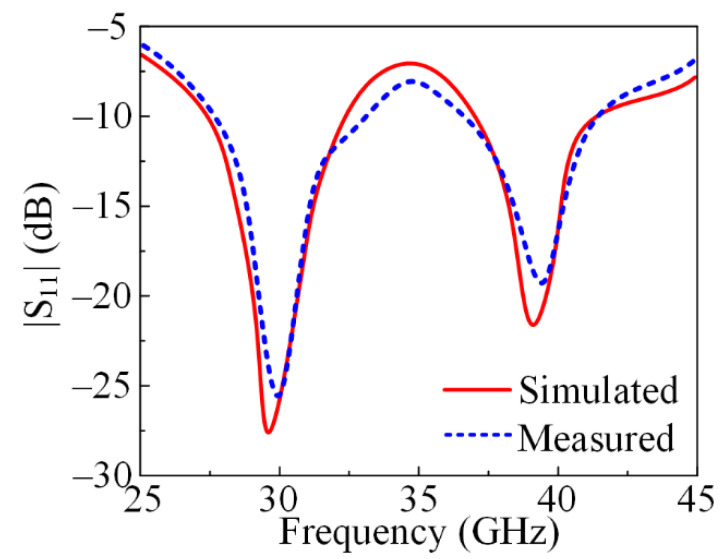
S-parameter plot of a proposed dual-band antenna operating at 28 GHz and 38 GHz, measured and simulated.

The suggested dual-band antenna’s measured and simulated S-parameters are shown in Figure 3. The antenna provides two bands: one at 28 GHz with an impedance bandwidth between 26 and 31.5 GHz, and another at 38 GHz with an impedance bandwidth between 36.5 and 41.7 GHz. Moreover, the figure shows the similarity between the S-parameters generated from software and the tested prototype, which makes the suggested antenna a good candidate for future 28/38 GHz applications. 

#### 2.3.2. Measured and Simulated Gain

**Figure 4:**
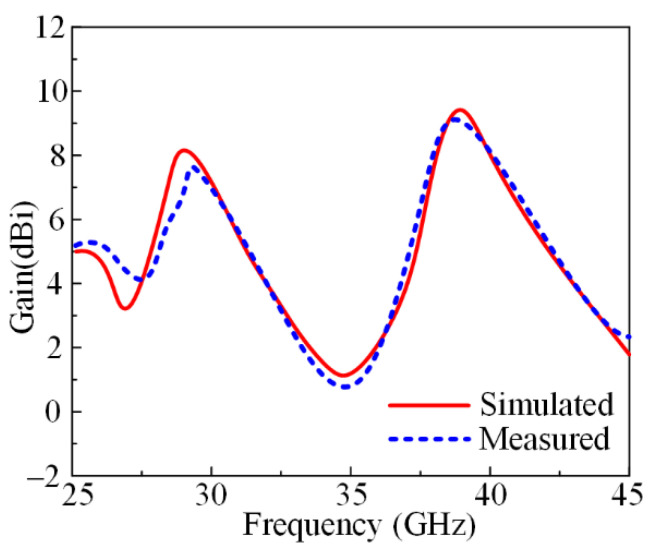
Measured and simulated gain of proposed dual-band antenna.

In Figure 4, the measured gain of the antenna prototype along with a comparison with the simulated gain is given. It can be noticed that the antenna offers a gain of >7 dBi at an operational bandwidth of 26–31.5 GHz, with a peak value of 8 dB at the resonance frequency of 28 GHz. Meanwhile, the antenna offers a gain of >8.25 dBi at an operational bandwidth of 36.5–41.7 GHz, with a peak value of 9 dB at the resonance frequency of 38 GHz. Moreover, the figure shows good settlement between the software-generated results and the hardware-measured results, which makes the proposed antenna a good and suitable candidate for future high-gain 5G applications. 

#### 2.3.3. Measured and Simulated Radiation Pattern

**Figure 5:**
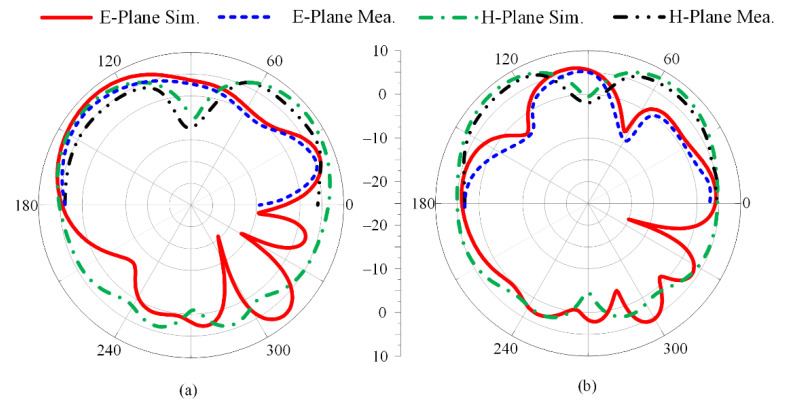
Measured and simulated radiation pattern of proposed dual-band antenna at (**a**) 28 GHz and (**b**) 38 GHz.

Figure 5 depicts the radiation pattern at resonance frequencies of 28 GHz and 38 GHz. The suggested wideband millimeter-wave antenna revealed a broadside radiation pattern in the theoretical E-plane (θ = 0°) in contrast to the butterfly-shaped pattern that was examined in the H-plane (θ = 90°). In general, there is good agreement between the measured and simulated E-plane and H-plane data for both resonance frequencies.

The suggested dual-band antenna is compared in Table 1 to previous antennas in the literature on the same frequency range. The overall size of the antenna, operational bandwidth, operating frequency, and gain are compared. The table shows that the suggested antenna is small in size compared to the others proposed in [8,9,12] and offers dual bands with a wide bandwidth and high gain. Although the antennae presented in [6,10,11] offer a compact size, they only have simulated results, which limits their useability in practical application. From the above results, discussion, and comparison with the literature, it is demonstrated that the suggested antenna is an excellent candidate for upcoming millimeter-wave applications.

## 3. MIMO Configuration of Proposed Antenna

### 3.1. MIMO Antenna Design Procedure

**Figure 6:**
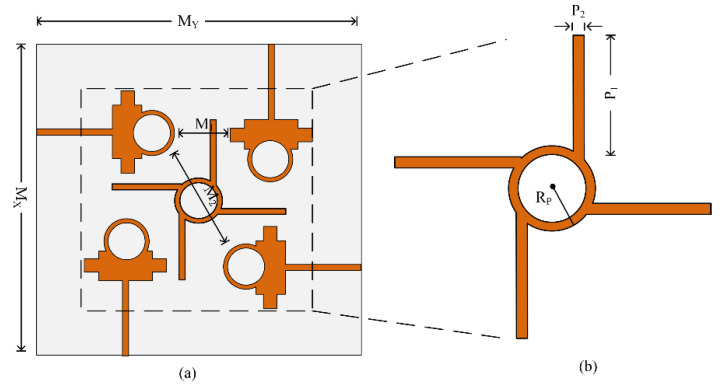
Proposed parasitic element loaded MIMO antenna: (**a**) top view; (**b**) parasitic element.

The proposed dual-band antenna’s MIMO arrangement for operation at 28/38 GHz is shown in Figure 6. The proposed MIMO antenna system contains four elements in an orthogonal pattern with a full ground plane on the backside. The MIMO system has an overall size of *M*_*X*_ × *M*_*Y*_ = 28 mm × 28 mm. The parasitic element is placed at the center of the antenna to reduce mutual coupling within the antenna, as given in Figure 6. The MIMO antenna system was designed by rotating the 90° antenna element on the *z*-axis. The size of the substrate was increased due to the MIMO configuration; the rest of the antenna parameters are the same as for the single antenna element discussed in Section 2 and given in Figure 1. The optimal parameters of the MIMO antenna and parasitic element are given as follows: *M_X_* = 28; *M_Y_* = 28; *M*_1_ = 4.65; *M*_2_ = 13.2; *P*_1_ = 6.4; *P*_2_ = 1.35; *R_P_* = 3.25 (all units are in millimeters (mm)).

**Figure 7:**
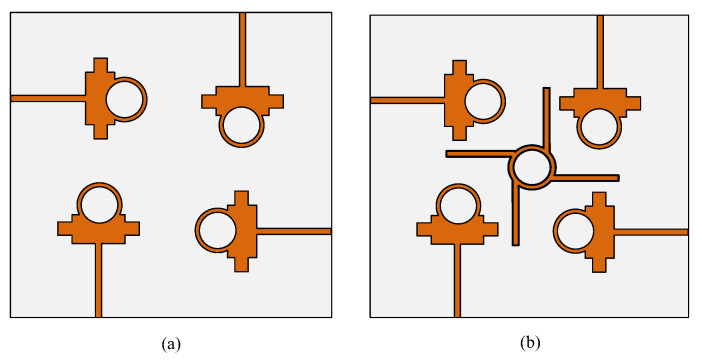
MIMO design evolution: (**a**) without parasitic element; (**b**) with parasitic element.

**Figure 8:**
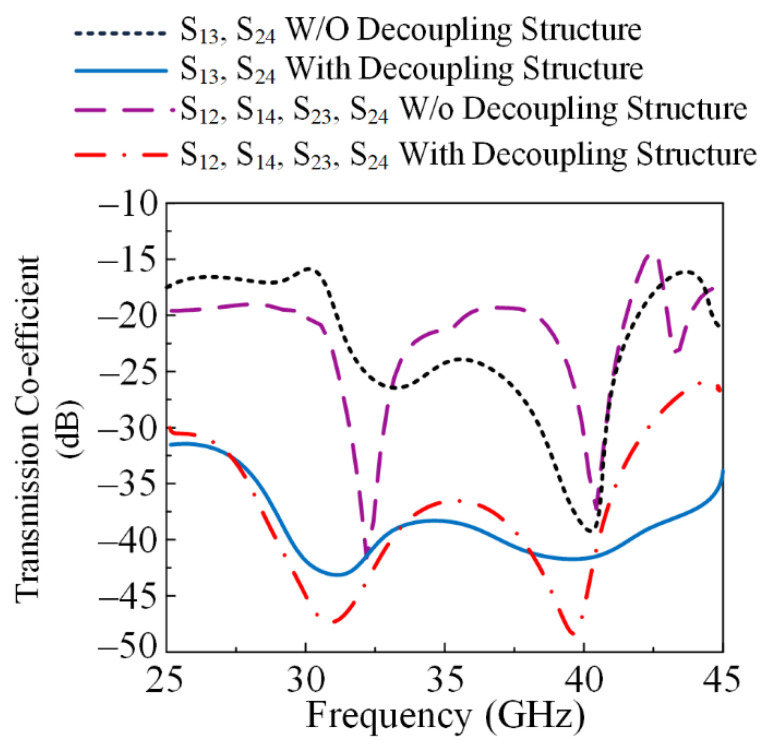
Comparison plot of transmission coefficient with and without parasitic element.

Figure 7 exhibits the four-port MIMO antenna system with and without the parasitic element, while the ground plane is unchanged in both cases. The parasitic element is introduced between elements of the MIMO system to overcome the amount of energy generated by nearby elements when another element radiates. This effect of one element of the MIMO system on another is also called mutual coupling. The reduction in mutual coupling can also be expressed by the following equations given in [29]:

(4) $$MC_{ij}=e^{\left(-\frac{2dij}{\lambda }\left(\alpha +j\pi \right)\right)}\;;i\neq j$$

(5) $$MC_{ij}=1-\frac{1}{N}\sum_{I}\sum_{i\neq j}d_{ij}$$

where *d_ij_* expresses the space between the ith and jth elements, *N* represents the number of array components, and α is the fine-structure constant having a value approximately equal to 0.00729735. The comparison of transmission coefficients in terms of the antenna effect by adjacent antenna elements (S_12_, S_23_, S_34_, S_41_) and diagonal elements (S_24_, S_31_) is given in Figure 8. The MIMO antenna system without a parasitic element offered a maximum value of −25 dB at 28 GHz and −36 dB at 40 GHz under the effect of diagonal elements. Meanwhile, under adjacent elements, the antenna offered a maximum of −40 dB at 32 GHz and −35 dB at 41 GHz. After placing the parasitic element, the antenna offered a minimum value <−30 dB for both cases, with maximum values of −45 dB and −50 dB at 31 GHz and 39 GHz, respectively. The comparison of mutual coupling with and without the parasitic element is given in Figure 8. 

### 3.2. MIMO Antenna Performance Parameter

**Figure 9:**
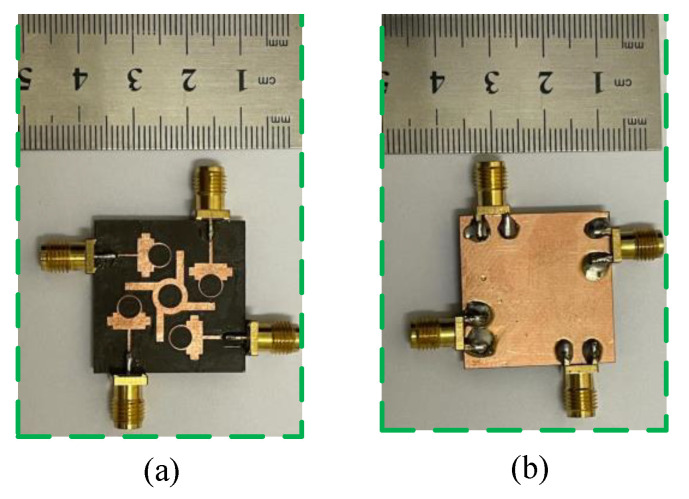
Hardware prototype of proposed parasitic element-loaded 4-port MIMO antenna for 28/38 GHz millimeter-wave applications (**a**) top side (**b**) bottom side.

To verify the simulated results generated by the software tool, a hardware prototype was fabricated, as given in Figure 9. To analyze and verify the transmission and reflection coefficient, the 220 ZVA Vector Network Analyzer (VNA) by Rohde & Schwarz was used. 

#### 3.2.1. Reflection Coefficient 

**Figure 10:**
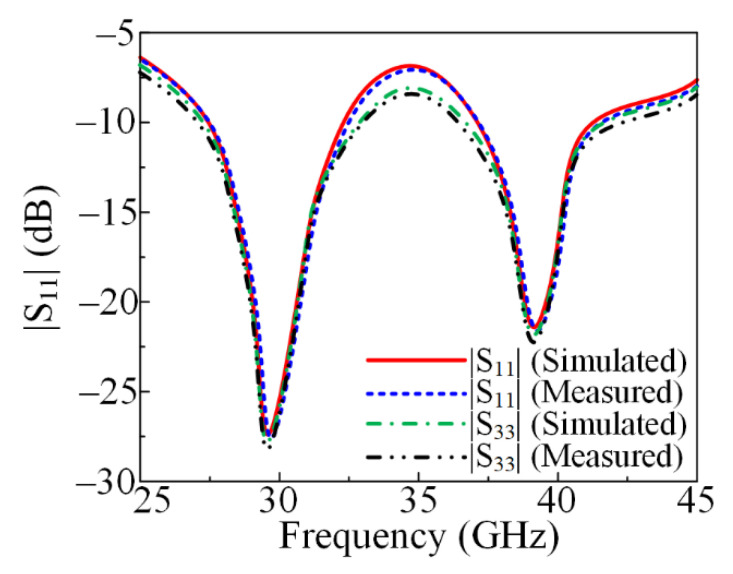
Comparison of measured and simulated reflection coefficients with parasitic element.

The simulated and hardware-measured reflection coefficients of the proposed parasitic element-loaded wideband antenna for 28/38 GHz applications are depicted in Figure 10. It can be observed that the proposed antenna offers dual bands at 28.5 GHz and 38.5 GHz having impedance bandwidths of 26.5–31.5 GHz and 36–41.7 GHz, respectively. The proposed parasitic element-loaded MIMO antenna operates at a low value of return loss <−20 dB for both resonance bands. Moreover, the simulated and measured results show strong agreement, with negligible changes. This similarity in the simulated and measured results and the dual wideband operation make the proposed antenna a suitable design for future 5G millimeter-wave applications. 

#### 3.2.2. Transmission Coefficient 

**Figure 11:**
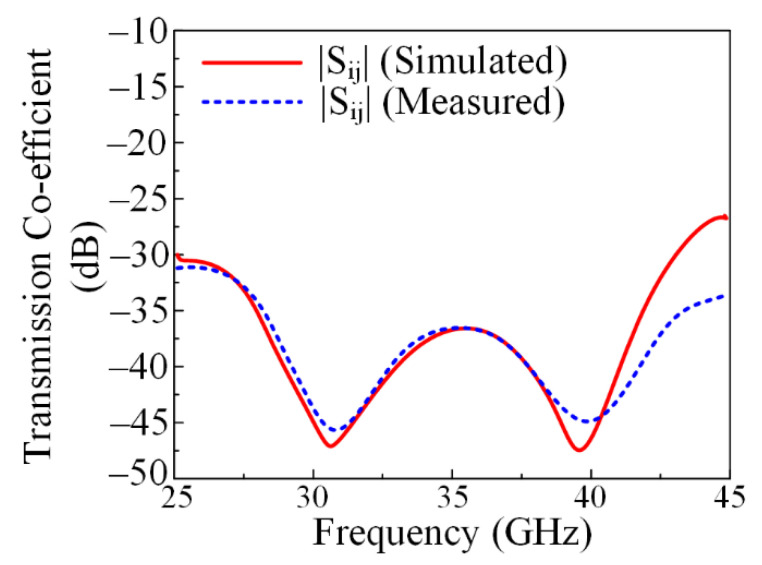
Comparison of measured and simulated transmission coefficients with parasitic element.

Figure 11 presents the measured and simulated transmission coefficients of the suggested parasitic element-loaded four-port MIMO system. It can be noticed from the provided figure that the antenna offers isolation of <−35 dB in the operational bandwidth region, with a minimum value of −48 dB at 31 GHz and 39 GHz. The results are provided for the antenna element with its nearby antenna and diagonal antenna for better understanding. The mutual coupling was reduced by placing the parasitic element between the MIMO antenna elements as discussed earlier. The simulated results show strong similarity with the measured results, which makes the proposed antenna a good candidate for future millimeter-wave devices. 

#### 3.2.3. Radiation Pattern

**Figure 12:**
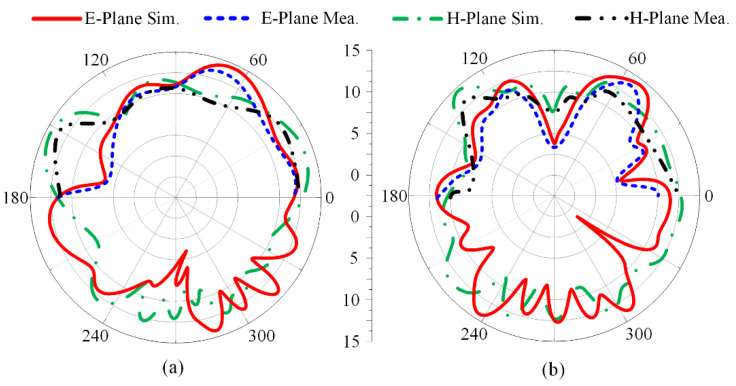
Measured and simulated radiation patterns of proposed parasitic element-loaded dual-band MIMO antenna at (**a**) 28 GHz and (**b**) 38 GHz.

The radiation pattern of the proposed parasitic element-loaded four-port MIMO antenna is given in Figure 12 at the resonance frequency bands of 28 GHz and 38 GHz. It can be noticed that the suggested antenna for mm-wave application provides a broadside radiation pattern at the principal E-plane and a slightly distorted radiation pattern at the H-plane. The distortion in the radiation pattern is due to multiple stub insertions and slots etched from the radiating patch. Moreover, similarity is observed between the measured and simulated results, which makes the proposed MIMO antenna system a potential and suitable applicant for 5G millimeter-wave application. 

#### 3.2.4. Envelop Correlation Coefficient (ECC)

**Figure 13:**
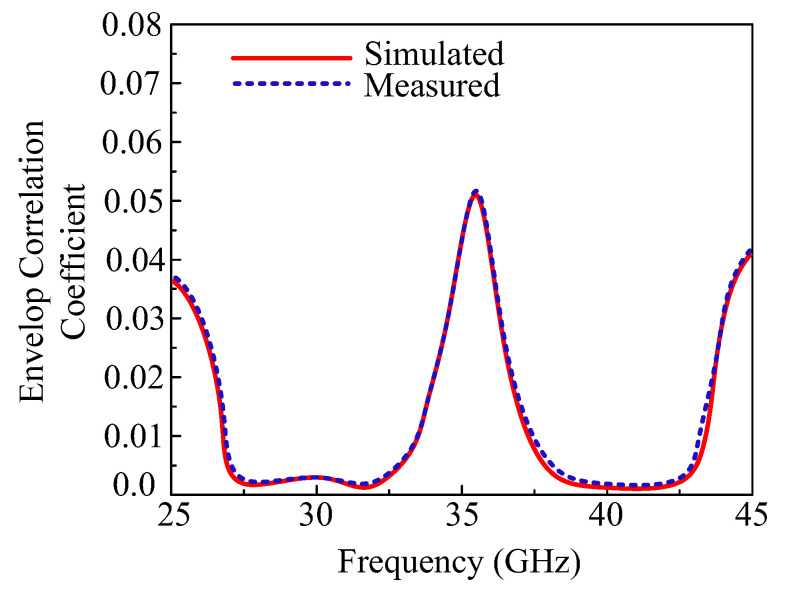
Envelop Correlation Coefficient (ECC) of the proposed parasitic element-loaded dual-band MIMO antenna.

The Envelop Correlation Coefficient (ECC) measures the performance of one antenna element. The ECC of the MIMO antenna can be measured in terms of the S-parameter and the far-filed radiation pattern. The ECCs of the adjustment element and diagonal element are given in Figure 13. The suggested antenna delivers an ECC < 0.001, as can be seen in the figure below, for both operational bandwidths in the case of the nearby antenna element and the diagonal element. The mathematical equations for measuring the ECC are given below [30]:

(6) $$\rho_{eij}=\frac{{\left|\iint_{0}^{4\pi }\left[\vec{R_{i}}\left(\theta ,\;\varphi \right)\times \vec{R_{j}}\left(\theta ,\;\varphi \right)\;\right]d\Omega \right|}^{2}}{\iint_{0}^{4\pi }{\left|\vec{R_{i}}\left(\theta ,\;\varphi \right)\right|}^{2}d\Omega \iint_{0}^{4\pi }{\left|\vec{R_{j}}\left(\theta ,\;\varphi \right)\right|}^{2}d\Omega }$$

The above equation expresses the general form to calculate the ECC, where *i* and *j* are the antenna elements of a MIMO system. 

#### 3.2.5. Diversity Gain (DG)

**Figure 14:**
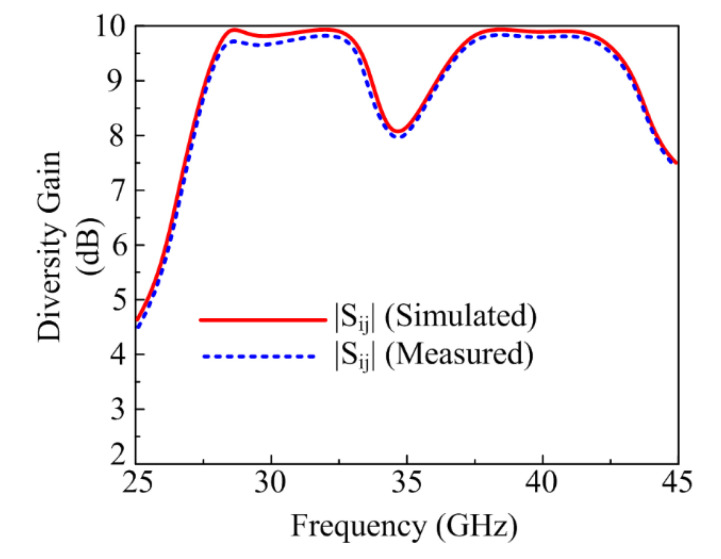
Diversity Gain (DG) of the proposed parasitic element-loaded dual-band MIMO antenna.

For the MIMO antenna system, when a diversity scheme is performed, losses occur in the form of transmission power. These losses were analyzed by studying Diversity Gain (DG). For an ideal scenario, DG must be equal to 10 dB, but for an actual scenario, values close to 10 dB are considered. The proposed antenna showed a DG around 9.99 dB for both the operational bands, as given in Figure 14. Mathematically, DG can be expressed as follows [30]:

(7) $$DG=10\;\sqrt{1-|ECC{|}^{2}}$$

#### 3.2.6. Channel Capacity Loss (CCL)

**Figure 15:**
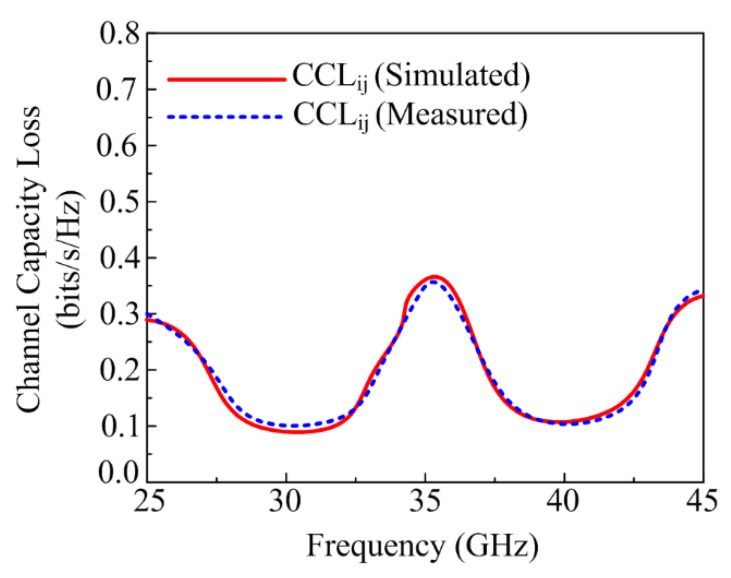
Channel Capacity Loss (CCL) of the proposed parasitic element-loaded dual-band MIMO antenna.

To check the performance of the MIMO antenna system, Channel Capacity Loss (CCL) is at the leading edge. CCL occurs in the MIMO system due to correlation losses. The proposed parasitic element-loaded four-port MIMO antenna offers around 0.01 bps/Hz for both operating bandwidths of 28 GHz and 38 GHz, as provided in Figure 15. The acceptable range of CCL is <0.5 bps/Hz, and the mathematical equation to calculate it is given below [31]:

(8) $$C_{Loss}=-log_{2}\;\det\left[\propto^{R}\right]$$

where

(9) $$\left[\propto^{R}\right]=\left[\begin{array}{cc} \propto_{11} & \propto_{12}\\ \propto_{21} & \propto_{22} \end{array}\right]\;\propto_{11}=1-\left({\left|S_{11}\right|}^{2}+{\left|S_{12}\right|}^{2}\right);\;\propto_{12}=S^{*}{}_{11}S_{12\;}+S^{*}{}_{21}S_{12};\;\propto_{21}=S^{*}{}_{22}S_{21\;}+S^{*}{}_{12}S_{21};\;\propto_{22}=1-\left({\left|S_{22}\right|}^{2}+{\left|S_{21}\right|}^{2}\right)$$

#### 3.2.7. Mean Effective Gain (MEG)

**Figure 16:**
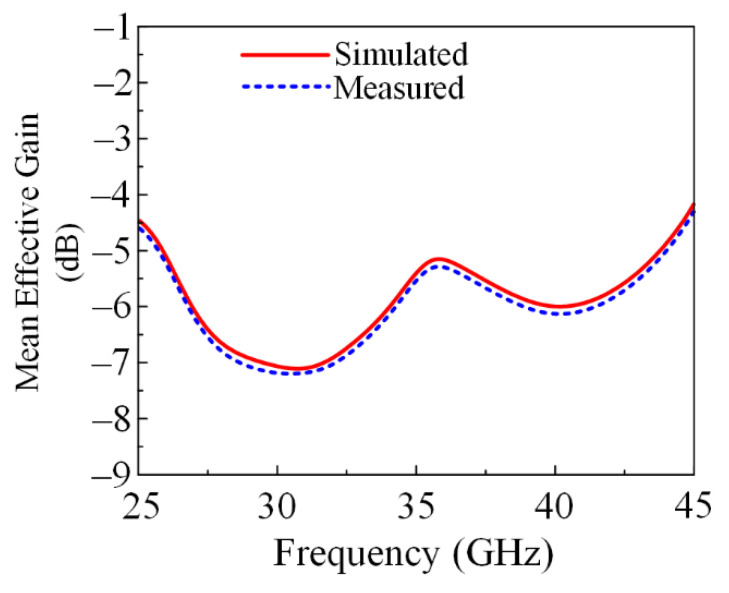
Mean Effective Gain (MEG) of the proposed parasitic element-loaded dual-band MIMO antenna.

Mean Effective Gain (MEG) represents the power received in a fading environment by any wireless system. For any MIMO antenna system, the fair range of MEG is <−3 dBi. The proposed antenna showed an MEG < −5.5 dBi at both operational bandwidths, as given in Figure 16. Moreover, the mathematical equation to calculate the MEG is given below [31]:

(10) $$\mathrm{MEG}=0.5\;\left[1-\sum_{i,j=1}^{n}\left|S_{ij}\right|\right]$$

In the above equation, $P_{\emptyset }$ represents the angular density function of incident power and XPR is the cross-polarization power ratio [32].

In Table 2, a comparison between the proposed four-port MIMO antenna and previous research is provided. From the below table, it is clear that the suggested MIMO antenna system has a compact size, offers wide dual bandwidths, offers high gain, and has reduced mutual coupling with the simple parasitic element shape. The comparison table, the discussion, and the results above indicate that the proposed antenna is a potential dominant candidate for future compact, high-gain, broadband 5G portable devices.

## 4. Conclusions

In this article, a four-port MIMO antenna loaded with a parasitic element has been presented. A rectangular patch antenna was initially created for 28 GHz applications. The rectangular patch antenna’s slot was engraved and various stubs were loaded to create a dual-band 28/38 GHz antenna with a large operational bandwidth and minimal return losses. Later on, configuration of a four-port MIMO was performed with a parasitic element to cut back mutual coupling among the four MIMO antenna elements. The parasitic element-loaded MIMO antenna has a compact size and simple geometry and operates over a wide bandwidth of 26.5–31.5 GHz with a resonance frequency of 28 GHz and 36–41.7 GHz with a resonance frequency of 38 GHz. Stringent agreement between the simulated and measured findings was seen. A hardware prototype was created to verify the simulated results. The MIMO parameters ECC, CCL, DG, and MEG were also studied to analyze the antenna. The parasitic element-loaded MIMO antenna had an ECC of <0.001, CCL of <0.01 bps/Hz, DG of around 9.99 dBi, and MEG of <−5.5 days. The existing literature was compared with the MIMO antenna results, which further indicated that the suggested antenna outperforms the related antennae, making it a strong candidate for future 5G applications.

## Figures and Tables

**Table 1:** Comparison of proposed dual-band antenna with the state of the art.

Ref.	Overall Antenna Size (mm × mm)	Operational Frequencies (GHz)	Antenna Type	Operational Bandwidth (GHz)	Gain (dBi)
[6]	5 × 5	28	Printed antenna	26.5–29	6.5
[8]	90.49 × 29.43	38	Array antenna	31–39	16.4
[9]	21 × 26	28/38	Enhanced Franklin antenna	24–30 34–40	4.2 6.9
[10]	5 × 5	28/38	Patch antenna	27.5–29 37–38.5	6.6 5.6
[11]	6.2 × 8.4	28	Patch antenna	26–31	3
[12]	12 × 18	28/38	Patch antenna	26–29 37–40	6.6 7
Prop. Design	13 × 15	28/38	Patch antenna	26–31.5 36.5–41.7	7 8.25

**Table 2:** Comparison of the proposed parasitic element-loaded MIMO antenna for isolation improvement with the state of the art.

Ref.	Overall Antenna Size (mm^3^)	Operational Frequencies (GHz)	Operational Bandwidth (GHz)	Max Isolation without Parasitic Element (dBi)	Max Isolation with Parasitic Element (dBi)	Gain (dBi)	Technique Adopted for Isolation Improvement
[22]	20 × 40 × 1.6	27	26.7–28.9	−−16	−29.3	5.6	Metamaterial structure
[23]	15.3 × 8.5 × 0.79	27/38	28.5–29.5 37.5–38.45	−52	−60	6.19 7.16	Double-sided EBG structure
[24]	20 × 20 × 2.794	28	27.5–28.5	−22	−34	8.2	Metallic strip
[25]	15 × 26 ×1.57	15	15.5–17	−25	−38	9.8	Parasitic element
[26]	25 × 15 × 1	28/38	26.5–31 36–40	−22	−44	6.2 5.8	DRA
[27]	5 × 57.5 × 0.508	24/28	23.3–25.2 26.9–29	−16 −17	−20 −24	18 16.02	Additional stub-loaded patch antenna
This work	28 × 28 × 0.79	28/38	26.5–31.5 36–41.7	−40	−50	9.5 11.5	Parasitic element

## Data Availability

Not applicable.
